# Mr. Ring, on the Vaccine Inoculation

**Published:** 1801-06

**Authors:** John Ring

**Affiliations:** New-street, Hanover-square


### *To the Editors of the Medical and Phyfical Journal.*

Gentlemen,

It will afford many of your readers no fmall degree of fatisfaction to hear, that Vaccine Inoculation is eftablifhed in the Eaft and Weft Indies, in Sicily, and in Ruffia.

By an over-land difpatch, lately arrived, we are informed it is practifed at Bombay; probably, by means of the matter fent from Conftantinople by Dr. Whyte; and it is eftablifhed at Jamaica by Mr. Rooke, Surgeon, at Montpelier, Montego Bay.

Mr. R. having inoculated himfelf and fifteen negroes with vaccine virus received from Dr. Croft, infection took place in himfelf alone. In confequence of the frequent failure of infection, in his own hands and thofe of others, Mr. R. began to fufpect that people of colour were infufceptible of the difeafe.

He

He is now well convinced that this fufpicion was unfounded. Neverthelefs the infertion of the vaccine fluid in his own arm was fingularly providential.

When the laft advices were received from Mr. R. he had fuccefsfully inoculated all the people on the eftate of Charles Rofe Ellis, Efq. except thofe who laboured under the yaws; and many of them had been fubmitted, both by effluvia and inoculation to the infection of the fmall-pox, which they refifted.

By thefe inconteftible proofs of its efficacy, the moft incredulous were convinced; and the practice was making a rapid progrefs throughout the ifland. The fmall-pox had been fatal to many eftates. In one, where it was making the moft dreadful ravages, it was foon checked by the introduction of the cow-pox.

By a letter from Dr. Marfhall, dated Palermo, March 27, 1801, I am informed, that he arrived in Sicily on the 12th of that month, and had commenced the vaccine inoculation with fuccefs. The King of Naples has appointed an hofpital, where his firft phyfician and firft furgeon are to inoculate, under the direction of Dr. Marfhall and Dr. Walker, and inftruct the other phyficians and furgeons of the country, that the practice may be eafily propagated and permanently eftablifhed in that kingdom.

At Peterfburg, Dr. Halliday has inoculated feveral perfons in the hofpital, with the fame happy fuccefs that has attended the practice in every other country.

I am, &c.

*New-ftreet, Hanover-fquare,*

*May* 20, 1801.

JOHN RING.

